# Discovering genomic islands in unannotated bacterial genomes using sequence embedding

**DOI:** 10.1093/bioadv/vbae089

**Published:** 2024-06-17

**Authors:** Priyanka Banerjee, Oliver Eulenstein, Iddo Friedberg

**Affiliations:** Department of Computer Science, Iowa State University, Ames, IA 50011, United States; Department of Computer Science, Iowa State University, Ames, IA 50011, United States; Department of Veterinary Microbiology and Preventive Medicine, Iowa State University, Ames, IA 50011, United States

## Abstract

**Motivation:**

Genomic islands (GEIs) are clusters of genes in bacterial genomes that are typically acquired by horizontal gene transfer. GEIs play a crucial role in the evolution of bacteria by rapidly introducing genetic diversity and thus helping them adapt to changing environments. Specifically of interest to human health, many GEIs contain pathogenicity and antimicrobial resistance genes. Detecting GEIs is, therefore, an important problem in biomedical and environmental research. There have been many previous studies for computationally identifying GEIs. Still, most of these studies rely on detecting anomalies in the unannotated nucleotide sequences or on a fixed set of known features on annotated nucleotide sequences.

**Results:**

Here, we present TreasureIsland, which uses a new unsupervised representation of DNA sequences to predict GEIs. We developed a high-precision boundary detection method featuring an incremental fine-tuning of GEI borders, and we evaluated the accuracy of this framework using a new comprehensive reference dataset, Benbow. We show that TreasureIsland’s accuracy rivals other GEI predictors, enabling efficient and faster identification of GEIs in unannotated bacterial genomes.

**Availability and implementation:**

TreasureIsland is available under an MIT license at: https://github.com/FriedbergLab/GenomicIslandPrediction.

## 1 Introduction

Horizontal gene transfer (HGT) in bacteria is an important mechanism for the acquisition of genetic material, enabling adaptation to a changing environment by rapidly conferring new phenotypes such as stress resistance and antibiotic resistance (Koonin *et al.* 2001, Thomas and Nielsen 2005). Genomic islands (GEIs) are clusters of genes acquired by HGT. Those can provide evolutionary diversity and, in addition, confer complex traits that require several gene products that are co-expressed (Hacker and Carniel 2001, Dutta and Pan 2002, Dobrindt *et al.* 2004, Boyd *et al.* 2009, Juhas *et al.* 2009). GEIs are typically classified based on their functional content: pathogenicity islands that contain pathogenic or virulent genes, resistance islands containing antimicrobial-resistance genes, symbiosis islands containing genes that establish symbiosis with host organisms, or metabolic islands containing adaptive metabolic capabilities [for a review, see: (Bertelli *et al.* 2019)]. GEIs have some distinguishing features, including (i) a typical size range of 10–200 kb (Hacker and Kaper 2000), (ii) a sequence composition that is generally different from that of the host genome, and (iii) frequent associations with tRNA-encoding genes, flanking direct repeats, and mobility genes, with a high prevalence of phage-related genes and hypothetical proteins (Dobrindt *et al.* 2004). The wide range of adaptive functions makes the identification of GEIs of particular environmental and biomedical interest (Hacker and Kaper 2000, Juhas *et al.* 2009). GEIs are experimentally identified using methods such as DNA–DNA hybridization, subtractive hybridization, or using counterselectable markers (Reyrat *et al.* 1998, Winstanley 2002, Dobrindt *et al.* 2004). However, experimental methods are limited to specific combinations of bacterial strains and GEI types and can be expensive and time-consuming. Therefore, reliable GEI prediction methods are needed. Methods for computationally predicting GEIs are broadly divided into two approaches: comparative genomics and sequence composition. Comparative genomic approaches involve the use of closely related bacterial and archaeal genomes (Bertelli *et al.* 2019). A GEI is identified when a cluster of genes that is not present in any related organisms is present in an organism (Langille *et al.* 2008). Recently, Bertelli *et al.* conducted research showing that comparative genomics-based approaches can accurately predict the boundaries of GEI. However, such methods depend on the availability of closely related organisms. Furthermore, results vary widely depending on the selected genomes and are often a time-consuming process (Langille *et al.* 2008, Bertelli *et al.* 2019). Sequence-composition methods are based on identifying atypical subsequences in the chromosome. These methods identify aberrations in structural features, such as GC content, dinucleotide content, codon usage, *k*-mer count, presence of various insertion sites, presence of mobility genes, phage genes, hypothetical proteins, and direct flanking repeats (Dobrindt *et al.* 2004, Juhas *et al.* 2009). One recent prediction method used deep learning to predict one such structural feature HGT insertion sites (Li *et al.* 2020). Prediction methods that only use sequence composition are usually less accurate than those using annotated sequences and comparative genomics. This is due to the limited information the sequence composition methods are working with (Bertelli *et al.* 2019). In sum, there are certain limitations to current GEI prediction methods: (i) the requirement of presence and correct selection of closely related organisms in case of comparative genomics-based approach; (ii) the comparative genomics-based approaches, though very accurate, are often the more laborious and time-consuming approaches, (iii) dependency on annotated genomes, and (iv) lack of a good feature set in a sequence composition-based approach. Here, we present TreasureIsland, a GEI prediction software that uses an unsupervised representation of DNA sequences and, therefore, does not require any computation of a fixed number of features. TreasureIsland is a fast and efficient tool that does not require annotated genomes, nor does it require any process of selection of closely related organisms for reference. Specifically, TreasureIsland uses document embedding for the detection of differential sequence compositions. Document embedding is an extension of the more popular word embedding models. These models are particularly powerful in natural language processing as they capture the semantic and syntactic quality of the documents upon which they are trained (Mikolov *et al.* 2013) and can be applied to DNA and protein sequences whose string representation is treated as documents. Word embedding has been used in several bioinformatics applications, including novel ORF identification (Hamid and Friedberg 2019), DNA origin of replication (Wu *et al.* 2021), assignment of function to protein domains (Buchan and Jones 2020), mapping the gut microbiome (Tataru and David 2020), and protein family classification (Asgari and Mofrad 2015). To our knowledge, this is the first time document embedding has been used to discover GEIs. Furthermore, the ability to identify genomic features in unannotated genomes using word embedding opens up many possibilities of genomic feature discovery in genomes and metagenomes.

## 2 Methods

### 2.1 Dataset construction

#### 2.1.1 The Benbow dataset

To train and validate our model, we needed a large, accurate, and non-redundant dataset of positive and negative GEIs. Therefore, to train TreasureIsland, we compiled data from four well-established GEI databases into a unified non-redundant dataset we named Benbow (Stevenson 1883) (Table 1). The main database (M) (Bertelli and Brinkman 2018) incorporates the L database (Langille *et al.* 2008) that includes negative examples from literature curation. The early (E) dataset is a curated dataset that was used to build IslandPath (Hsiao *et al.* 2003). Finally, we added non-redundant sequences from PAIdb (P) (Yoon *et al.* 2015) to create the full database of positive and negative examples as elaborated.

**Table 1.** Sources of GEIs used to construct Benbow, the unified GEI set used in this study.^a^

Notation	Total genomes	Number of GEI	Number of non-GEI	Data source
M (Main)	104	1845	3266	Bertelli and Brinkman (2018)
E (Early)	32	269	0	Hsiao *et al.* (2003)
L (Literature)	6	80	179	Langille *et al.* (2008)
P (PAI)	111	264	0	Yoon *et al.* (2015)
Benbow (M + E + P)	173	2004	3030	–

a See text for details.

Benbow consists of GEI regions (positive labels), which we call *BENBOW_pos_*, and non-GEI regions (negative labels), which we call *BENBOW_neg_*.

##### 2.1.1.1 Genomes in BENBOW


$$\begin{array}{c} \;\mathrm{BENBO}W_{\mathrm{pos}}:=M_{\mathrm{pos}}\cup E\cup P\\ \;\mathrm{BENBO}W_{\mathrm{neg}}:=M_{\mathrm{neg}} \end{array}$$

To construct *BENBOW_pos_* GEI regions we added: (1) GEIs from all genomes in *M* (104 genomes, containing 1845 GEI regions); (2) GEIs from all genomes in *E* that do not overlap with the genomes in *M* (32−8=24 genomes, containing 172 GEI regions); and (3) GEIs from all genomes in *P* that do not overlap with the genomes in *M* and *E* (111−22=89 genomes, containing 177 GEI regions). The combined dataset gives us a total of 1, 845 (*M* dataset) + 172 (*E* dataset) + 177 (*P* dataset) = 2194 GEIs. This dataset has an unequal distribution of organisms from different phyla. Since most phyla have no or very limited labeled GEI data, we limit *BENBOW_pos_* and *BENBOW_neg_* to *Pseudomonadota* and *Firmicutes*. Thus, the size of the total *BENBOW_pos_* dataset after eliminating any phyla other than *Pseudomonadota* and *Firmicutes* is 2, 004 GEIs. The total *BENBOW_neg_* dataset after the same elimination is 3, 030 non-GEIs.

To remove redundancy and reduce bias, we ran CD-HIT (Fu *et al.* 2012) using an 80% sequence identity cut-off on the positive and negative label datasets. This resulted in positive and negative examples of 1742 and 1393 regions, respectively, as shown in Supplementary Fig. S1.

##### 2.1.1.2 Benbow DNA-embedding model and machine learning dataset

Since our model aims to predict GEIs within a bacterial genome sequence context, we created the training and test datasets from whole genomes rather than use “disembodied” positive and negative GEI and non-GEI regions. To create the test dataset, we randomly selected 20 genomes, with 413 GEIs and 153 non-GEIs (a total of 566), and for training, we used 1329 GEIs from 145 genomes and 1240 non-GEIs from 72 genomes for a total of 2569 regions. We trained the document-embedding model and the machine learning model on the training data (see Supplementary Fig. S1). To tune the parameters in the machine learning models, we used 10× cross-validation. More details on hyperparameter tuning are provided in Section S3 of the Supplementary Material. TreasureIsland is currently limited to predicting from the taxonomic range covered in the Benbow dataset as shown in Supplementary Table S17.

### 2.2 Computational framework

**Figure 1.**
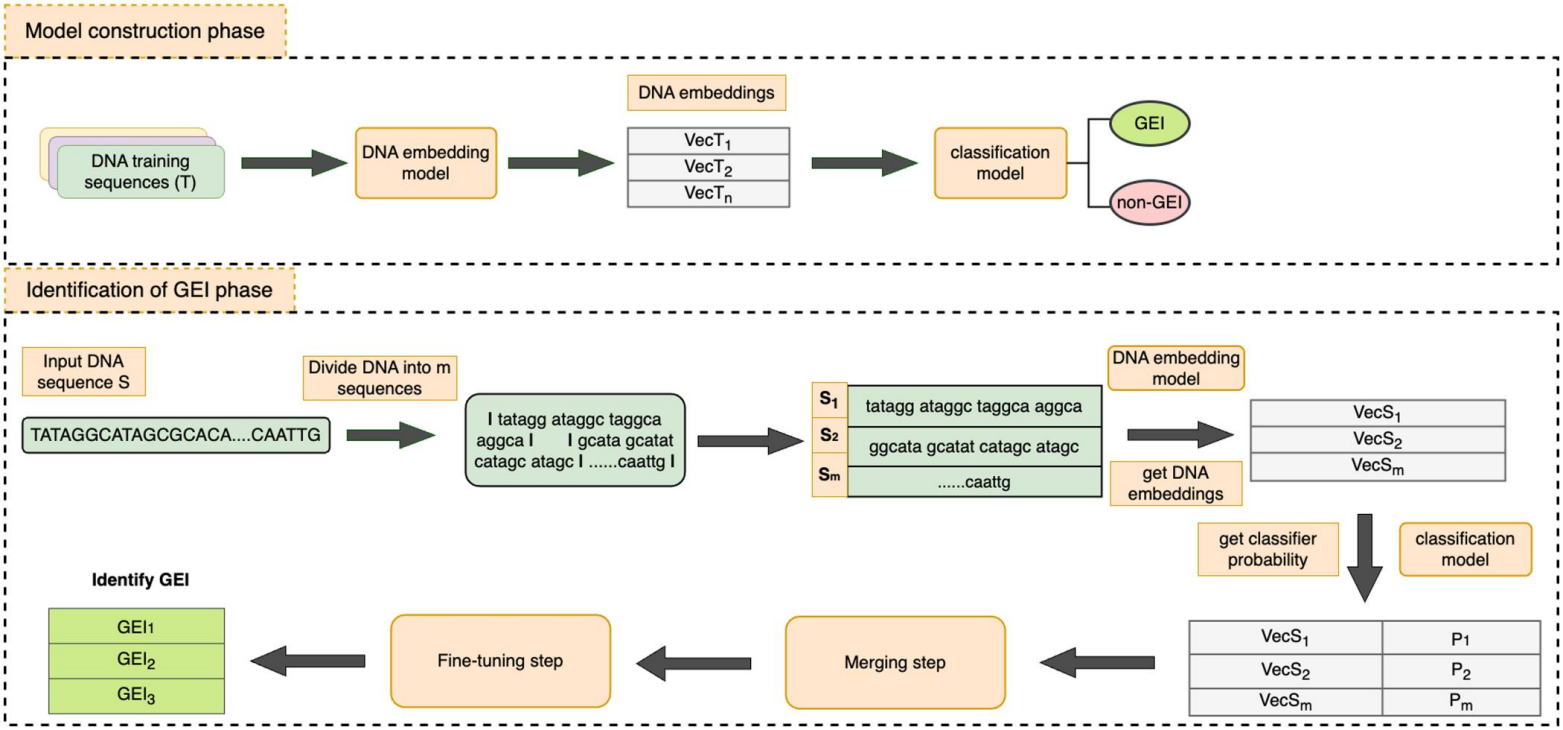
An overview of TreasureIsland. The TreasureIsland workflow consists of two phases: (i) The model construction phase for classifying DNA segments to GEI or non-GEI; this includes Doc2vec embedding of DNA sequences, and training a classifier. (ii) Identification of GEI location in the bacterial chromosome, including the fine-tuning of the GEI borders.

The TreasureIsland computational framework consists of two phases: (1) the model construction phase for classifying DNA segments as GEI or non-GEI and (2) GEI identification for any input sequence, typically a whole bacterial chromosome. As seen in the overview in Fig. 1, in the model construction phase, we construct an embedding model, which represents the variable-length DNA by fixed-length vectors. We then use these vectors to classify the segments of DNA into a GEI or a non-GEI region in a genome. At the end of the first phase, we are left with an embedding model and a classifier for DNA segments.

In the second phase, identification of GEIs, we divide an input sequence into non-overlapping segments (Supplementary Table S1, sequence_window_size). These segments are then embedded and classified using the embedding and classifier models from the model construction phase. The GEI-classified segments are then processed to refine the boundaries to output the GEI regions in the input DNA sequence.

#### 2.2.1 Phase 1: Model construction

Here, we construct the embedding and classifier models for DNA sequences.

##### 2.2.1.1 Background

After successfully implementing word2vec for basic word embeddings, researchers have tried to extend the same idea to vectorize multiple words in a sentence, paragraph, or even a document Le and Mikolov (2014) (here, we use paragraph and document interchangeably). Even though the weighted average of word vectors or bag of words models were simple solutions, this did not capture the word order. Doc2vec is an extension of the word2vec model proposed by Le and Mikolov (2014), converting a variable-length sentence to a fixed-length vector. Similar to the word2vec models, doc2vec models are designed as a classification task for predicting a word from the context of a document. Each document is mapped to a vector that is uniquely identified by a document ID. There are two different types of document models—distributed memory (DM) and distributed bag of word (DBOW).

In the DM model, a document ID is added as another word to the document’s words. The context of a training sample is selected by sliding a window of a specific size over the document. The model then averages or concatenates the document ID vector and the word vectors to predict the next word in the context. The vectors are then trained by stochastic gradient descent *via* backpropagation, and eventually, the model learns the word vectors and the document ID vector, representing the document. This model is analogous to the Continuous Bag of Words Model (CBOW) model in word2vec.

In contrast, DBOW is a much simpler and lighter model that trains only the document ID vector. This model ignores the order of words and predicts a randomly sampled word from the context given the document ID. This process is analogous to the skip-gram model in word2vec.

##### 2.2.1.2 DNA as a document

As noted in Section 1, word embedding has been successfully adopted in bioinformatics to classify biological sequences in many applications. Doc2vec is an extension of the word2vec model (Le and Mikolov 2014) that converts a variable-length document into a fixed-length vector. Each document is identified by a document ID, and is subsequently converted to a vector to represent every document.

##### 2.2.1.3 Embedding DNA sequences

**Figure 2.**
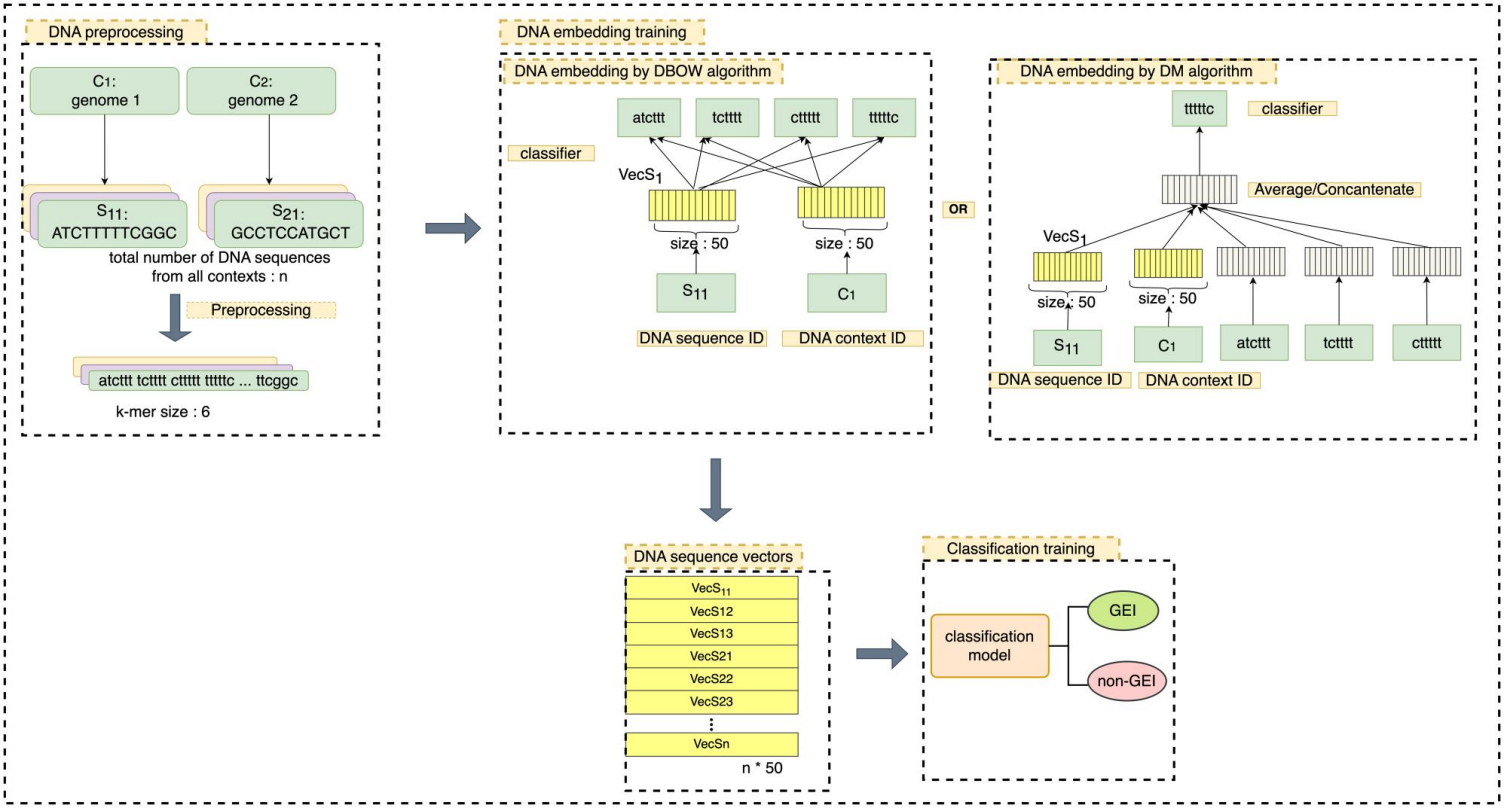
Model construction phase. Each DNA sequence in the training or validation set was pre-processed and then converted into a fixed-length vector using either the distributed bag of words (DBOW) or the distributed memory (DM) algorithm. These DNA vectors were then classified into GEI or non-GEI.

In our implementation to classify sequences, any sequence (GEI or non-GEI) is considered a document and is represented as a sequence of overlapping *k*-mers, which are the words. As shown in Fig. 2, each GEI and non-GEI example (length 1300–65 000 bp each), which contains *k*-mers as its words, is tagged by a unique DNA sequence ID. To represent the genome containing each GEI and non-GEI example, we add another tag of DNA context ID to all the training examples. This tag is identical for all GEI and non-GEI examples derived from the same genome. We then train the DM and DBOW models. At the end of the embedding model training, we converted the DNA sequence and context IDs from the training samples to a fixed-length vector.

##### 2.2.1.4 Constructing the classifier

After we trained the embedding model, we obtained the vectors for the training set and test set by using gradient descent from the embedding model while fixing the rest of the model parameters as in (Le and Mikolov 2014). We then fed the training vectors into several different supervised machine learning algorithms to complete a binary classification task as GEI (Class 1) or non-GEI (Class 0).

#### 2.2.2 Phase 2: Identifying genomic islands

This phase takes as an input a DNA sequence, usually a whole chromosome, and identifies all possible GEIs in the sequence. The DNA-embedding model and the classifier from the first phase are used here. Table 2 explains the parameters used for this phase.

**Table 2.** Parameters used for the GEI identification phase.

Parameter	Notation	Description	Value
DNA_sequence	*D*	Input DNA sequence	–
sequence_window_size	*W_s_*	Window size of the initial non-overlapping sequence	10 kb
kmer_size	*k*	*k*-mer size	6 bp
minimum_gi_size	*GEI_m_*	Minimum size of a GEI	10 kb
tune_window_size	*W_t_*	Window size for border tuning of a GEI	1 kb
upper_threshold	*T_u_*	Threshold of a segment above which a segment is classified as GEI	0.80
lower_threshold	*T_l_*	Threshold of a segment below which a segment is classified as non-GEI	0.50

##### 2.2.2.1 DNA vector representation

We divide the chromosome sequence *D* into *n* non-overlapping fixed-length segments. $D=[d_{1},d_{2},d_{3},\ldots ,d_{n}]$, where each segment is analogous to a document. We then preprocess each segment (“document”) in the same way as in the model construction phase by finding all *k*-mers of size *k*. We then embed the segments by inferring vectors from the DNA-embedding model. Finally, we feed the vectors into the classifier and determine the probability *p_i_* of any given segment *d_i_* to be a GEI where:

$$p_{1},p_{2},p_{3},\ldots ,p_{n}\;\text{with}\;p_{i}\in (0,1]$$

##### 2.2.2.2 Merging

**Figure 3.**
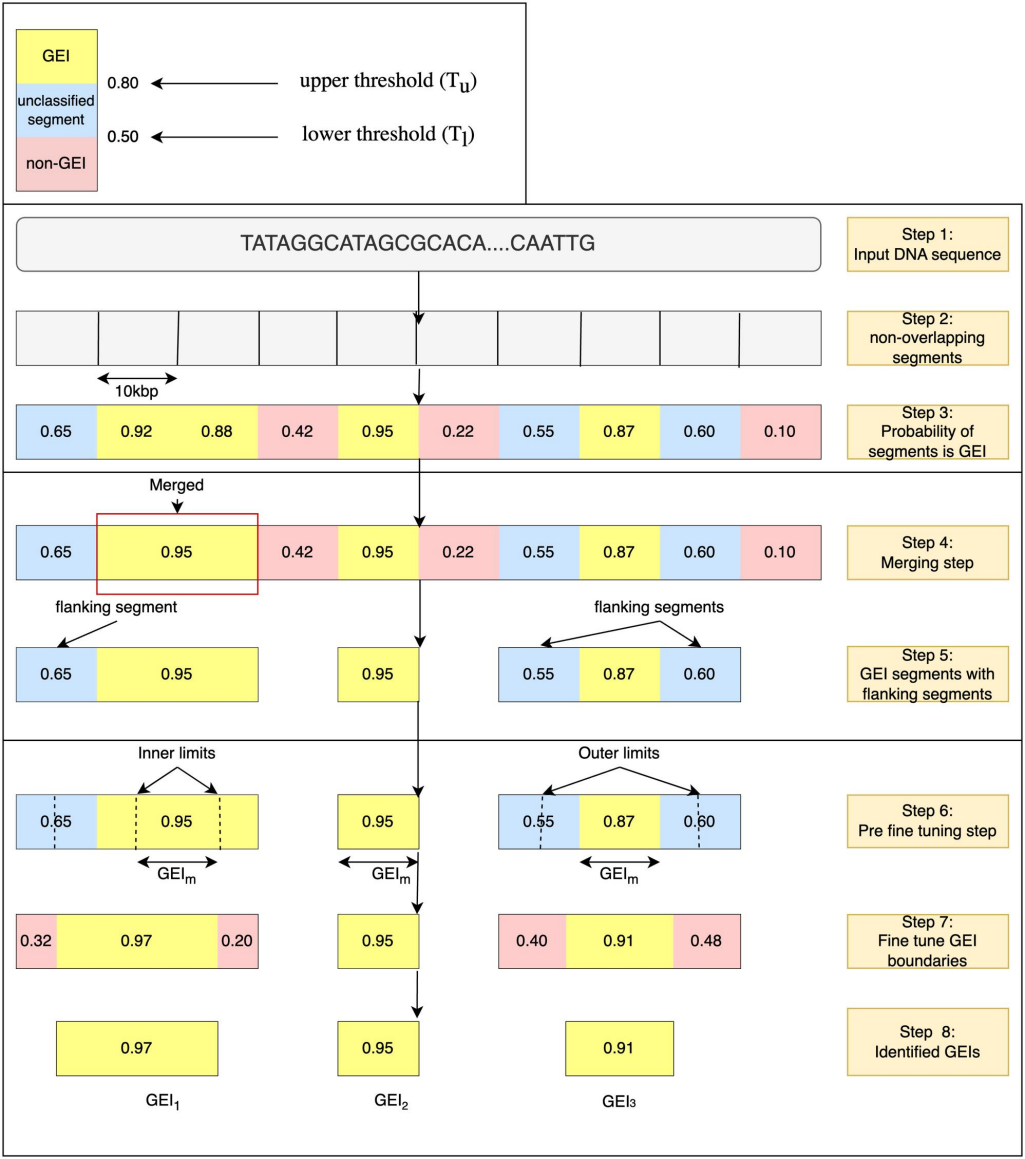
Identification of GEI phase. In this example, *T_u_* is set to 0.80 and *T_l_* is set to 0.50. The input DNA sequence is divided into non-overlapping DNA segments. The probabilities of each of the segments being a GEI are determined. Next, the adjacent positive GEIs are merged, and the unclassified segments are attached. These regions are then fine-tuned to find the final GEIs.

In the merging step, we merge adjacent segments identified as GEIs, where appropriate, as shown in Fig. 3 (see Algorithm 1). We set two GEI probability thresholds to determine merging: an Upper Threshold *T_u_* and a Lower Threshold *T_l_*. Any segment with a predicted GEI probability *P*(*GEI*) having $P(\mathrm{GEI})\geq T_{u}$ is labeled as a GEI. If two or more adjacent segments are found to be greater than or equal to *T_u_*, the segments are merged, and the merged sequence is considered to be a GEI (see Fig. 3, Step 4, red box). Segments with $P(\mathrm{GEI})\leq T_{l}$ are labeled non-GEI. We considered the segments having probabilities between *T_u_* and *T_l_* unclassified since we are unsure which class they belong to. Therefore, to identify a more precise border, the unclassified segments are subject to a fine-tuning algorithm (Algorithm 1) to eliminate them and refine the GEI/non-GEI classification. The fine-tuning algorithm iteratively increases the probability of each segment being a GEI by changing the start and end positions of the segments.

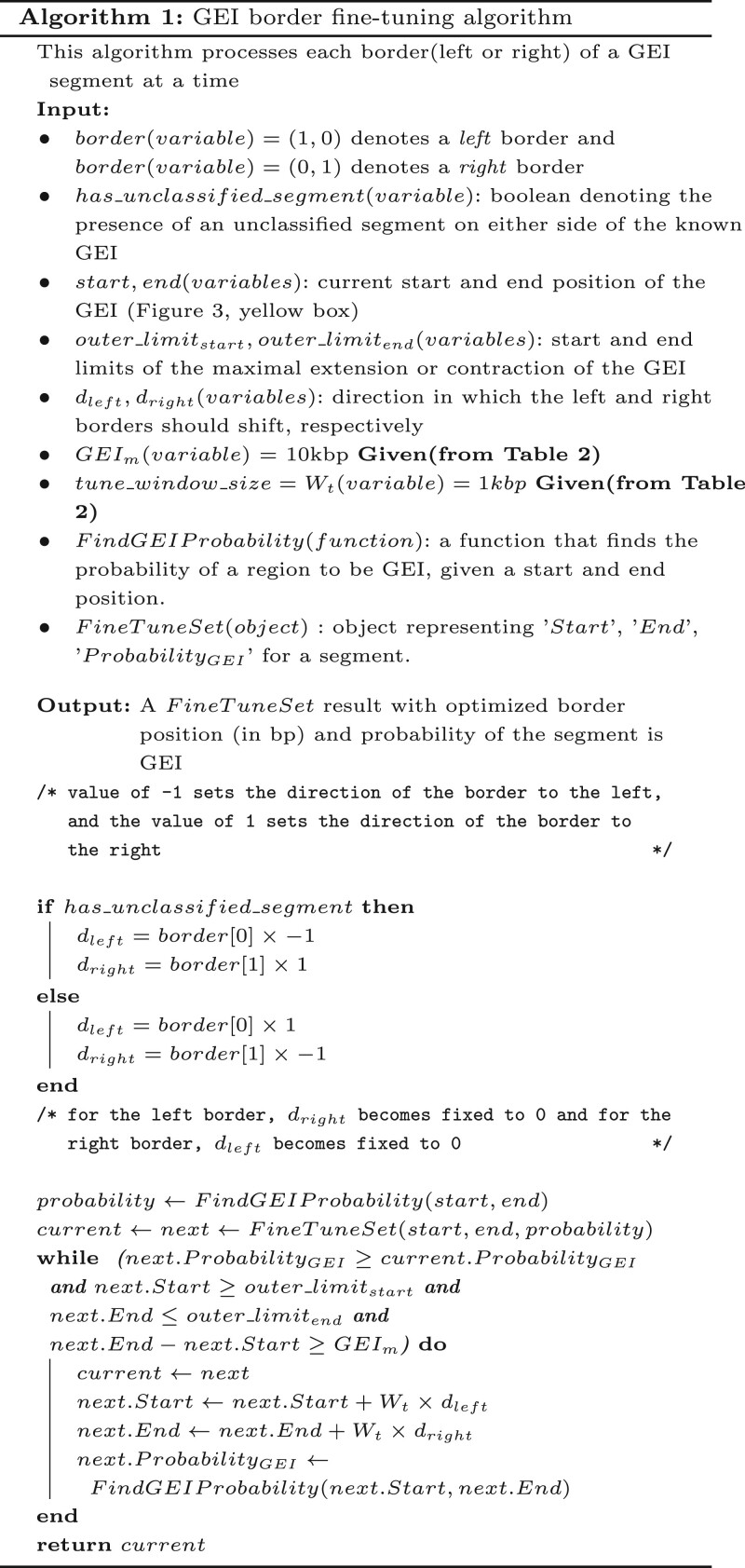

### 2.3 Evaluating TreasureIsland’s performance

Here, we evaluated the model’s performance for computational resources and accuracy and compared its performance with other models.

#### 2.3.1 Model evaluation for computational resources and accuracy

To evaluate TreasureIsland’s performance, we first examined the performance of different combinations of embeddings and classifiers. All experiments have been conducted on a Macbook with a M1 chip, 8 cores and 16 GB RAM. We used Gensim’s Doc2vec package in Python to find the document vectors using both DM and DBOW algorithms. We also used Gensim’s ConcatenatedDoc2Vec package in Python for the concatenated DM + DBOW model. For the baseline model TFIDF, we used Gensim’s Dictionary and TfidfModel packages in Python. First, we trained the embedding models distributed memory (DM), distributed bag of words (DBOW), concatenated DM and DBOW model (DM + DBOW), and Term Frequency–Inverse Document Frequency (TFIDF), on the training dataset. The training dataset was created using 145 genomes with a total of 2569 regions, and is 40 MB in size.

The training dataset was then vectorized for each of the Doc2vec DNA-embedding models: DBOW, DM, and DM + DBOW using gradient descent from the Doc2vec inference step. The machine learning dataset vectors were also found for the baseline embedding model TFIDF. Table 3 shows the total training time required and the vector dimension used for each embedding model. TFIDF exhibited the fastest training time, but it required the largest space for storing the vectors for any *k*-mer length above 3. The DM + DBOW model combined the vectors generated by both the DM and DBOW models. Thus, the total training time for this model aggregates the training time for DM and DBOW. Also, DM and DBOW models required the least storage space for their vectors.

**Table 3.** Comparison of vector dimension and training time among embedding models DBOW, DM, DM + DBOW, and TFIDF trained on the same training data with a *k*-mer length *k*.

Model	Vector dimension	Training time (wall-clock min)
DBOW	50	30
DM	50	70
DM + DBOW	100	100
TFIDF	$4^{k}$	20

We formulated the task as a binary classification task, where the labels 1 and 0 represent GEI and non-GEI, respectively. We used the machine learning classifiers that are most commonly seen associated with document classification tasks, such as support vector machine (SVM), logistic regression (LR), and *k*-nearest neighbor (KNN). To tune the hyperparameters, we used 10× cross-validation grid search results. The computational time for all the above-mentioned machine learning models remained less than 10 min of wall-clock time. The evaluations of the classifiers were done based on their overall accuracy, precision, recall, and *F*_1_-score (the harmonic mean of the precision and recall). The classification task also helped to evaluate the performance of the different DNA-embedding models. More information on hyperparameter tuning is available in Supplementary Material S3. To better understand the impact of training on datasets of different sizes, we trained and tested the DBOW method on *Firmicutes* only, and on pooled *Firmicutes* and *Pseudomonadota*. We then performed *t*-SNE dimension reduction to visualize vector space and separation between the GEIs and non-GEIs. The results shown in Supplementary Figs S3 and S4 indicate that training on the pooled and more extensive dataset comprising both phyla provides better separation between the vectors.

#### 2.3.2 Comparing TreasureIsland with other methods

Here, we compare the predictions from TreasureIsland with other GEI prediction models that have previously shown good results: a tool with high precision based on detecting tRNA fragments (Islander), sequence composition-based tools (IslandPath-DIMOB, Sigi-HMM, AlienHunter), and a hybrid tool (IslandViewer4) (Vernikos and Parkhill 2006, Waack *et al.* 2006, Hudson *et al.* 2015, Bertelli *et al.* 2017, Bertelli and Brinkman 2018). The reference dataset used for this task is assembled from 20 genomes from the *M* dataset and six from the L dataset. A list of the test genomes is available in Supplementary Material.

## 3 Results and discussion

### 3.1 Model evaluation

**Figure 4.**
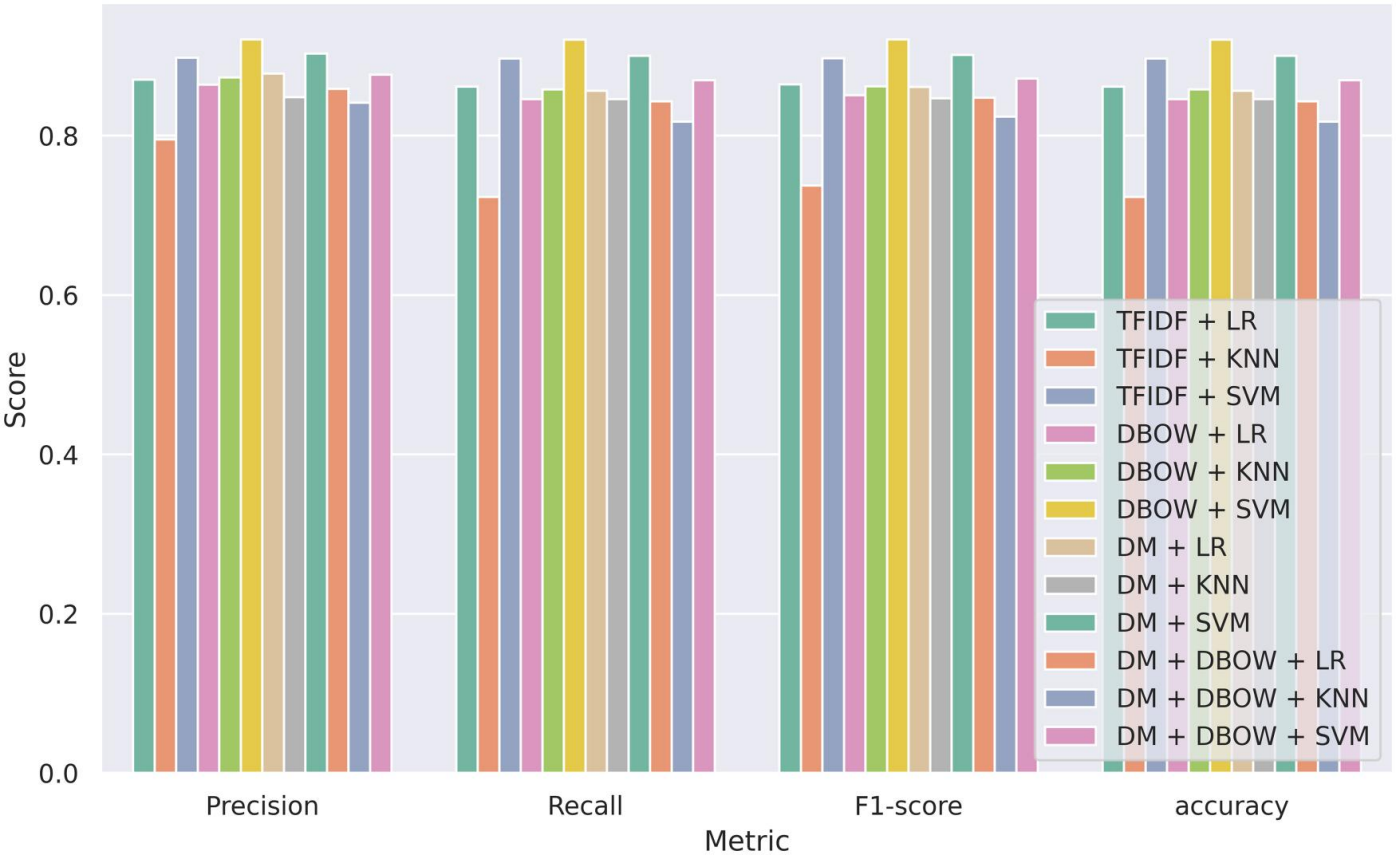
Weighted averaged precision, recall, *F*_1_-score, and accuracy for paragraph vector model distributed bag of words (DBOW) and other baseline representations term frequency–inverse document frequency (TFIDF), distributed memory (DM) model and concatenated DM and DBOW model (DM + DBOW) on classifiers logistic regression (LR), support vector machine (SVM), *k*-nearest neighbor (KNN). The full results are available in Supplementary Material.

**Figure 5.**
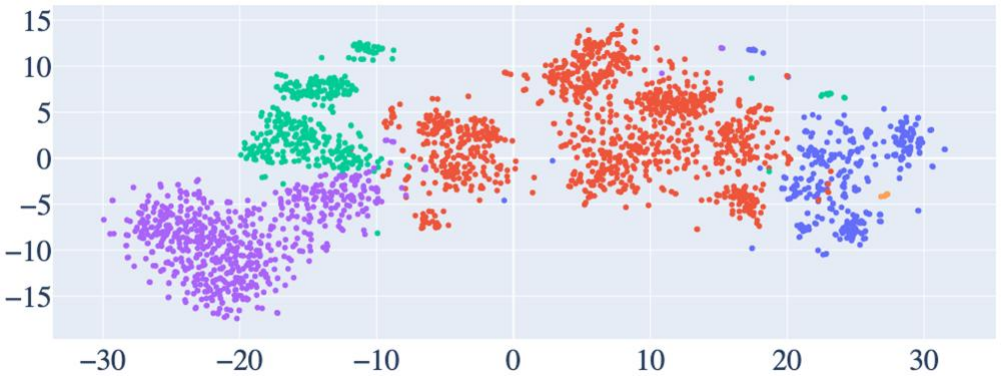
t-SNE (t-distributed Stochastic Neighborhood Embedding) visualization of the embedding training data vectors of the DNA sequence IDs from the DBOW model showing the distribution of different taxonomies. Blue (20 – 30 on X-axis, −10 – +5 on Y-axis): bacillus; orange (X: 0 – 20; Y: −5 – +15): clostridia; red (X: −10 – 0) *γ*-proteobacteria; green (X: −20 – −10; Y: 0–15): *α*-proteobacteria; purple (X: −30 – −10; Y: 20 – 0) *β*-proteobacteria.

**Figure 6.**
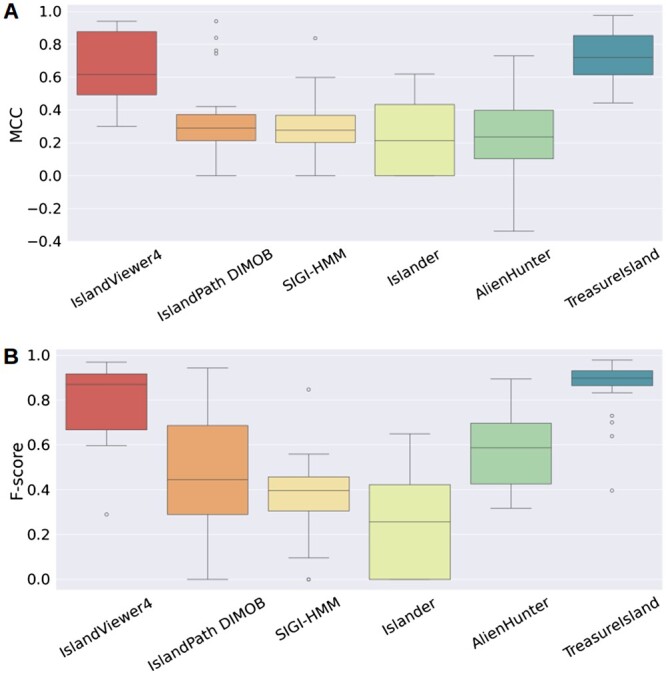
Box plot showing the distribution of performance on 20 genomes from comparative genomics test data across different models. (A) Distribution of MCC values. (B) Distribution of *F*_1_-score values.

Figure 4 shows that the DBOW + SVM model has the highest precision, recall, *F*_1_-score, and accuracy. Overall, SVM seems to perform best among all other classifiers in Figure 4 Although DBOW + SVM performs the best in the classification task, it is interesting that the TFIDF + SVM model also performs well in the classification task, showing that word relevance might indeed be a good factor for DNA embedding. We suspect that results from TFIDF that are sometimes comparable with the DBOW embedding may be attributed to the relatively small vocabulary size of *k*-mers, resulting in a limited number of unseen *k*-mers when trained with sufficient data. The Doc2vec DM embedding performs comparably with the DBOW model. It is important to note that the DBOW model, in addition to being the best-performing model, is also the lightest in terms of its size. Overall, we can see that SVM performs the best among all the classifiers. Therefore, we selected the DBOW + SVM model for the TreasureIsland software to perform further analyses. Figure 5 shows the *t*-SNE (*t*-distributed Stochastic Neighborhood Embedding) visualization of the embedding training data vectors of the DNA sequence IDs from the DBOW model. Broadly, it can be seen the different taxonomic classes in the embedding training data are separated in the vector space. Supplementary Figure S4 shows the vector space highlighting the GEI versus non-GEI sequence vectors from the DBOW model. Supplementary Figure S5 shows the *t*-SNE visualization of the DNA context IDs for each genome used in the training data from the DBOW model. While the *t*-SNE outcomes do not definitively confirm the separation of Taxonomic groups in the vector space, the figures strongly suggest that the DBOW model can learn meaningful taxonomic information from the DNA sequences (see Fig. 6).

### 3.2 Evaluation of GEI identification

Any nucleotide sequence longer than or equal to the minimum GEI size *GEI_m_* can be used to identify the GEI regions. However, since a typical input would be a whole bacterial chromosome, we used those for the evaluation. The complete list of genomes and results are available in the Supplementary Material.

We used standard metrics to assess the GEI predictors for the evaluation (Bertelli *et al.* 2019). The following values were identified based on nucleotide overlaps:

(i) True Positive (TP): the number of nucleotides in the positive prediction that overlaps with positive reference data. (ii) True Negative (TN): the number of nucleotides outside the positive prediction that overlaps with negative reference data. (iii) False Positive (FP): the number of nucleotides in the positive prediction that overlaps with negative reference data. (iv) False Negative (FN): the number of nucleotides outside the positive prediction that overlaps with positive reference data.

Based on these values, the following evaluation metrics are used:

$$\begin{array}{c} \text{Precision}=\frac{TP}{TP+FP};\text{Recall}=\frac{TP}{TP+FN};\\ F_{1}=2\times \frac{\text{precision}\times \text{recall}}{\text{precision}+\text{recall}};\text{Accuracy}=\frac{TP+TN}{TP+FP+TN+FN};\\ MCC=\frac{TP\times TN-FP\times FN}{\sqrt{\left(TP+FP)\times (TP+FN)\times (TN+FP)\times (TN+FN\right)}} \end{array}$$

#### 3.2.1 Comparative performance on the main (M) dataset

This experiment gives us a good idea about the potential of TreasureIsland to predict GEIs from an input sequence, especially the GEI identification phase, when using the models from the first phase. Note that the method performance shown in Tables 4 and 5 are similar to those shown in Bertelli *et al.* (2019), verifying the veracity of this analysis.

**Table 4.** Precision, recall, accuracy, *F*_1_-score, and MCC (Mathews correlation coefficient) achieved by TreasureIsland and other baseline GEI predictors on the Benbow test set.^a^

Predictor	Precision	Recall	Accuracy	*F*_1_-score	MCC
TreasureIsland	0.87 ± 0.04	0.88±0.02	0.90±0.02	0.86±0.03	0.75±0.03
IslandViewer4	0.93 ± 0.03	0.73 ± 0.05	0.83 ± 0.03	0.80 ± 0.04	0.66 ± 0.05
IslandPath DIMOB	0.91 ± 0.03	0.38 ± 0.06	0.64 ± 0.04	0.48 ± 0.06	0.35 ± 0.06
SIGI-HMM	0.98 ± 0.01	0.24 ± 0.04	0.60 ± 0.05	0.36 ± 0.04	0.30 ± 0.04
Islander	**1.0**	0.16 ± 0.04	0.56 ± 0.06	0.25 ± 0.05	0.22 ± 0.05
AlienHunter	0.70 ± 0.05	0.55 ± 0.04	0.65 ± 0.04	0.58 ± 0.04	0.26 ± 0.06

a Mean values ± standard error are shown in the table entries.

**Boldface** values are maximum of each method.

**Table 5.** Precision, recall, accuracy, *F*_1_-score, and MCC (Mathews correlation coefficient) achieved by TreasureIsland and other baseline GEI predictors on six genomes from the L dataset.^a^

Predictor	Precision	Recall	Accuracy	*F*_1_-score	MCC
TreasureIsland	0.96 ± 0.01	0.91±0.02	0.93±0.01	0.93±0.01	0.86±0.02
IslandViewer4	1.0	0.67 ± 0.04	0.81 ± 0.02	0.79 ± 0.02	0.68 ± 0.03
IslandPath DIMOB	1.0	0.48 ± 0.03	0.70 ± 0.02	0.64 ± 0.03	0.53 ± 0.02
SIGI-HMM	**1.0**	0.20 ± 0.04	0.55 ± 0.01	0.31 ± 0.05	0.27 ± 0.04
Islander	**1.0**	0.23 ± 0.03	0.56 ± 0.02	0.35 ± 0.04	0.3205 ± 0.02
AlienHunter	0.75 ± 0.07	0.57 ± 0.06	0.70 ± 0.02	0.64 ± 0.07	0.40 ± 0.07

a Mean values ± standard error are shown in the table entries.

**Boldface** values are maximum of each method.

Additional analysis depicted in Supplementary Fig. S2 highlights the influence of adjusting *T_u_* on the model’s performance. *T_u_* values ranging between 0.80 and 0.90 illustrate the trade-off between TP Rate and FP Rate, while striving to maintain optimal performance in the upper-left corner of the figure.

#### 3.2.2 Comparative performance on the literature (L) dataset

Table 5 shows the results for the analysis of the curated literature dataset. The predictors display a higher precision in this analysis, which means the TP rate has increased since the FP rate is held constant with the same negative dataset (from M, the only dataset that has verified true negatives). TreasureIsland shows an improved performance on the literature dataset with the highest *F*_1_-score, accuracy, and recall.

## 4 Conclusion

Here, we presented TreasureIsland, a document-embedding learning-based framework for predicting GEIs in unannotated bacterial DNA. TreasureIsland takes unannotated nucleotide sequences as input and uses an unsupervised representation of DNA to classify the GEI and non-GEI regions. We introduce a novel boundary refinement technique to designate GEI regions more accurately. Finally, we provide a new database of GEIs, Benbow, for training other methods.

We show that TreasureIsland has high recall, accuracy, and a precision comparable to some of the current baseline predictors. Due to its high recall, TreasureIsland has the potential to discover novel GEI regions that other predictors have not covered. We also introduce a novel model for the unsupervised representation of DNA, which can be helpful in other DNA-based predictions that use machine learning. TreasureIsland has shown improved performance over the current state-of-the-art methods, and since it does not require any gene annotations, we can use this method on newly sequenced unannotated genomes to predict GEI regions. We have also performed a time analysis in Supplementary Table S16 to find TreasureIsland to be the fastest-performing method among the models that take unannotated sequences as input.

However, since TreasureIsland does not use prior information such as gene components in its features, it may over-predict GEIs. These predicted GEIs might fall into the grey zone where we do not know if a region is truly a GEI or a FP. This problem relates to the broader open-world problem in computational biology: the difficulty of obtaining negative training data or ascertaining the veracity of proposed negative training data (Dessimoz *et al.* 2013, Jiang *et al.* 2014). While solutions to the problem have been proposed in other ML applications (Bendale and Boult 2015, Lakkaraju *et al.* 2017), it is still an open problem in many biological applications. At this time, TreasureIsland is limited by the data representations in Benbow and, therefore, loses performance predicting GEIs from taxa not in Benbow. Consequently, we have introduced a check in the software to verify if a genome is represented. Despite these constraints, unsupervised representations of the DNA are a powerful way of understanding GEIs and, by extension, genomes. We expect that as more experimental data about GEIs comes in, the scope of TreasureIsland will be expanded to more taxa, and so will its accuracy.

Finally, TreasureIsland demonstrates how word embedding can be used to discover genome features in unannotated genomes. TreasureIsland can discover genome-wide features without relying on gene and regulatory motif discoveries. This is especially important when analysing unannotated novel bacterial genomes and MAGs—metagenomic assembled genomes—where genome annotation is nonexistent or might be misleading. We hope that TreasureIsland will be useful not only by itself but also that others can adopt our study to create useful models that will discover genome-wide features other than GEIs.

## Supplementary Material

vbae089_Supplementary_Data

## Data Availability

The datasets generated during the study are available at: https://doi.org/10.6084/m9.figshare.25513897.v1. The source code used in this study is available at: https://github.com/FriedbergLab/GenomicIslandPrediction
