# Supplementary material for: Discovering genomic islands in unannotated bacterial genomes using sequence embedding
Source: Bioinform Adv. 2024 Jun 17;4(1):vbae089. doi: 10.1093/bioadv/vbae089 (PMC11193100; doi:10.1093/bioadv/vbae089)
Supplement: vbae089_Supplementary_Data [file vbae089_supplementary_data.pdf]

Supplementary Material for "Discovering genomic islands in unannotated bacterial genomes using sequence embedding"

Priyanaka Banerjee, Oliver Eulenstein, Iddo Friedberg

S1 DNA embedding

S1.1 Background

To make use of the power of machine learning techniques, biological sequence data must be converted to a form that can be understood by the machines. Previously, the popular method of embedding DNA data was to use a one-hot encoding. Given the volume of DNA data, one hot encoding becomes an expensive technique. With the advance in NLP techniques of word embedding, DNA embedding methods improved as well. One of the first papers on converting DNA to vector using a word embedding model from Natural Language Processing (NLP), by Patrick Ng 2017, showed the effectiveness of DNA2vec in numeric operations such as concatenation and assessing global alignment similarity. For instance, let us consider an operation using the Nearest-Neighbor algorithm [3].
Nearest-Neighbor($\overrightarrow{AAC}+\overrightarrow{TCT}$) $\in \{AACTCT, TCTAAC\}$ and, $\overrightarrow{ACGAT}-\overrightarrow{GAT}+\overrightarrow{ATC} \approx \overrightarrow{ACATC}$
Here, the neighbors of the 3-mers AAC and TCT when added overlap with their string concatenation AACTCT. The second equation shows the result of the nucleotide concatenation. Thus, the importance of representing DNA as vectors is demonstrated.

Natural Language Processing (NLP) has explored many word embedding techniques in recent years as it is a vital preprocessing step for all machine learning tasks. Some of these techniques, such as Word2vec, where words are converted to vectors, have been found to be very powerful as they capture the semantic meaning and the context of the words[2]. It is a neural network that uses both target words and context words to convert a word into a fixed-length vector. The vocabulary is built from the corpus and fed into the model. The word2vec model has two different variants known as Continuous Bag of Words (CBOW) and Skip-Gram. The CBOW learns representations by using the context word to predict the target word. It is a supervised learning algorithm with context words as input and a target word as output. The Skip-Gram model learns representations by using the target word to predict the context words. It is a supervised learning algorithm with a target word as input and context words as output.

S1.2 Term Frequency - Inverse Document Frequency (TFIDF)

TFIDF (Term Frequency - Inverse Document Frequency) is a widely used document embedding technique, which captures how important a word is to a document in a corpus. The TFIDF value increases proportionally to the number of times a word appears in the document, but is offset by the frequency of the word in the corpus, which helps to control for the fact that some words are generally more common than others. Intuitively TFIDF captures the word relevance in a particular document. It is calculated as the product of the term frequency and the inverse document frequency. The term frequency component measures the frequency of a word in a document. To

calculate the term frequency, we find the count of words in a document and normalize this count by the number of words in a document. To calculate the inverse document frequency, we first calculate the document frequency which can be found by counting the number of documents a word is present in. Inverse document frequency intuitively gives the informativeness of a word. IDF is calculated by the ratio of the total documents to the document frequency of a word. IDF values are low for highly occurring words. The logarithm of the IDF keeps the IDF value from exploding [4].

Given a document collection D, a word w, an individual document d ϵ D and total count of documents N

tf(w,d) = count of w in d / number of words in d

df(w) = occurrence of w in D documents

idf(t) = N/df(w)

$$tf - idf = tf(t, d) \times \log idf(t)$$

S2 Dataset Construction

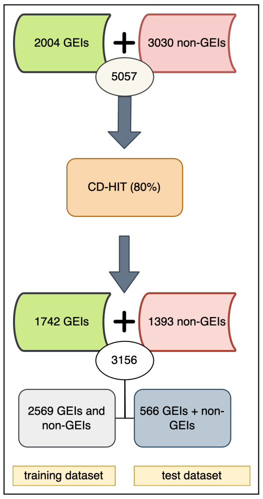

Figure S1: Creating the dataset for the model construction phase. The initial set of GEIs and non-GEIs was run through CD-HIT at 80% similarity to reduce redundancy and bias in the dataset. The machine learning test data was formed by separating 20 genomes with 566 regions from a total of 165 genomes. This left us with a document embedding and a machine learning training set of 2569 regions from 145 genomes.

S3 Hyperparameter tuning

S3.1 Tuning hyperparameters for the model construction phase

The hyperparameters include the k-mer length of $k \in \{3, \ldots, 9\}$ and the choice between overlapping and non-overlapping k-mers. At the document embedding level, the hyperparameters are tuned by using a grid search specific to each model. For both DBOW and DM models, the common hyperparameters include the vector size of the document, window size of the context of a document, epoch, alpha values, or the initial learning rate of the gradient descent algorithm. DBOW specifically was also tuned to dbow_words for training the word vectors along with the paragraph vectors. Similarly, the DM model was specifically tuned to dm_concat to determine whether to use concatenation or averaging vectors. The hyperparameters for the classification task were obtained using a 10× cross-validated grid search. The best choice for the document embedding model was the DBOW model, and the optimal hyperparameters were found to be k size of 6 with overlapping k-mers, vector size 50, window 15, epochs 150, alpha 0.025, dbow_words 0. The best classifier was found to be SVM, optimal at C=10, gamma = 1, kernel= RBF.

S3.2 Hyperparameters for the GEI identification phase

The parameters required to identify the GEIs in the fine-tuning step are described in Table 2. Those include: `window_size`, `kmer_size`, `minimum_gi_size`, `tune_window`, `upper_threshold` (T_u), and `lower_threshold` (T_l). These hyperparameters were obtained after tuning the identification of GEI results on the 70 training genomes containing positive and negative regions by grid search. The grid search results were found to be optimal with `window_size`=10,000, `kmer_size`=6, `minimum_gi_size`= 10,000 in keeping with the previous research on genomic island sizes [1], `tune_window`=1,000, `upper_threshold` (T_u)=0.80, `lower_threshold`=0.5.

S4 Results

Classifier	Precision	Recall	F1-score	Accuracy
**TF-IDF + LR**	0.8700	0.8614	0.8642	0.8614
**TF-IDF + KNN**	0.7950	0.7228	0.7374	0.7228
**TF-IDF + SVM**	0.8977	0.8965	0.8970	0.8965
**DBOW + LR**	0.8640	0.8456	0.8505	0.8456
**DBOW + KNN**	0.8728	0.8579	0.8619	0.8579
**DBOW + SVM**	**0.9207**	**0.9205**	**0.9206**	**0.9205**
**DM + LR**	0.8778	0.8561	0.8612	0.8561
**DM + KNN**	0.8483	0.8456	0.8468	0.8456
**DM + SVM**	0.9028	0.9000	0.9010	0.9000
**DM + DBOW + LR**	0.8587	0.8428	0.8473	0.8428
**DM + DBOW + KNN**	0.8411	0.8175	0.8239	0.8175
**DM + DBOW + SVM**	0.8764	0.8693	0.8716	0.8693

Table S1: Classification results on the test dataset. Weighted averaged precision, recall, f1-score and accuracy for paragraph vector model Distributed Bag of Words (DBOW) and other baseline representations Term Frequency–Inverse Document Frequency (TF-IDF), Distributed Memory (DM) model and Concatenated DM and DBOW model (DM+DBOW) on classifiers Logistic Regression (LR), Support Vector Machine (SVM), K-Nearest Neighbour (KNN).

Classifier	Mean Accuracy	Standard deviation
**TF-IDF + LR**	0.895540	0.020310
**TF-IDF + KNN**	0.830480	0.030459
**TF-IDF + SVM**	0.914201	0.013926
**DBOW + LR**	0.840720	0.022330
**DBOW + KNN**	0.887256	0.012581
**DBOW + SVM**	**0.928293**	**0.010383**
**DM + LR**	0.839260	0.021494
**DM + KNN**	0.854482	0.015977
**DM + SVM**	0.911852	0.015457
**DM + DBOW + LR**	0.855195	0.026295
**DM + DBOW + KNN**	0.859755	0.015128
**DM + DBOW + SVM**	0.908772	0.019878

Table S2: Mean accuracy and standard deviation for 10-fold cross-validation on different document models Term Frequency–Inverse Document Frequency (TF-IDF), Distributed Bag of Words (DBOW), Distributed Memory (DM) model and Concatenated DM and DBOW model (DM+DBOW) and classifiers Logistic Regression (LR), Support Vector Machine (SVM), K-Nearest Neighbour (KNN).

**K-mer size**	**Window method**					
	**overlapping window**			**non-overlapping window**		
	LR	SVM	KNN	LR	SVM	KNN
3	0.8402	0.9098	0.8234	0.7833	0.8961	0.7831
4	0.8204	0.9127	0.8341	0.7737	0.8981	0.8510
5	0.8213	0.9113	0.8611	0.8146	**0.9125**	0.8503
6	**0.8505**	**0.9206**	**0.8619**	**0.8272**	0.8972	**0.8628**
7	0.8140	0.9068	0.8614	0.7255	0.8666	0.8092
8	0.8276	0.9063	0.8608	0.7995	0.8428	0.7883
9	0.8501	0.8698	0.7931	0.7937	0.7458	0.6930

Table S3: Weighted F1-score of different k-mer sizes on classifiers Logistic Regression (LR), Support Vector Machine (SVM), K-Nearest Neighbour (KNN) for the model DBOW. Each k-mer model was tuned on document vector hyperparameters.

	Precision	Recall	Accuracy	F-Score	MCC
**mean**	0.933717	0.734717	0.834982	0.796522	0.657407
**std**	0.116702	0.21843	0.124127	0.170532	0.210641
**min**	0.513264	0.168848	0.597821	0.288913	0.299477
**25%**	0.901653	0.597587	0.75401	0.667868	0.49182
**50%**	0.989323	0.77037	0.835314	0.861072	0.616865
**75%**	1	0.914692	0.950143	0.916904	0.87671
**max**	1	1	0.992239	0.970321	0.94042

Table S4: Statistical summary of Precision, Recall, Accuracy, F-score, and MCC on 20 comparative genomics test data for IslandViewer.

	Precision	Recall	Accuracy	F-Score	MCC
**mean**	0.907358	0.381645	0.639215	0.481971	0.350233
**std**	0.154329	0.26848	0.199823	0.259982	0.269731
**min**	0.405393	0	0.392388	0	0
**25%**	0.886331	0.17172	0.481751	0.289372	0.214282
**50%**	1	0.293178	0.574249	0.431866	0.291436
**75%**	1	0.523385	0.799498	0.687073	0.363428
**max**	1	1	0.992239	0.942797	0.94042

Table S5: Statistical summary of Precision, Recall, Accuracy, F-score, and MCC on 20 comparative genomics test data for IslandPath dimob.

	Precision	Recall	Accuracy	F-Score	MCC
**mean**	0.983033	0.244746	0.597557	0.360418	0.30356
**std**	0.043663	0.182064	0.211454	0.196732	0.192392
**min**	0.827649	0	0.345538	0	0
**25%**	1	0.179631	0.424589	0.304553	0.200623
**50%**	1	0.247666	0.541788	0.396918	0.275746
**75%**	1	0.284694	0.765465	0.441801	0.368341
**max**	1	0.869269	0.97784	0.847948	0.836298

Table S6: Statistical summary of Precision, Recall, Accuracy, F-score, and MCC on 20 comparative genomics test data for SIGI HMM.

	Precision	Recall	Accuracy	F-Score	MCC
**mean**	1	0.164525	0.562247	0.247745	0.220403
**std**	0	0.174149	0.257751	0.24642	0.220502
**min**	1	0	0.052352	0	0
**25%**	1	0	0.374257	0	0
**50%**	1	0.14723	0.540512	0.256073	0.212304
**75%**	1	0.267688	0.803908	0.422187	0.432833
**max**	1	0.481898	0.949407	0.65038	0.619206

Table S7: Statistical summary of Precision, Recall, Accuracy, F-score and MCC on 20 comparative genomics test data for Islander.

	Precision	Recall	Accuracy	F-Score	MCC
**mean**	0.700199	0.546291	0.648169	0.579496	0.264732
**std**	0.216337	0.202548	0.177922	0.168888	0.282387
**min**	0.223971	0.224784	0.373073	0.316796	-0.337241
**25%**	0.556855	0.409395	0.510223	0.42517	0.102764
**50%**	0.738231	0.523192	0.617979	0.586409	0.236198
**75%**	0.861424	0.696729	0.818902	0.697683	0.398181
**max**	1	0.938382	0.953903	0.894233	0.729798

Table S8: Statistical summary of Precision, Recall, Accuracy, F-score and MCC on 20 comparative genomics test data for Alien Hunter.

	Precision	Recall	Accuracy	F-Score	MCC
**mean**	0.867049	0.882943	0.897013	0.856239	0.745622
**std**	0.196734	0.106105	0.069640	0.153519	0.146905
**min**	0.198655	0.607381	0.742020	0.331463	0.379347
**25%**	0.799822	0.838457	0.860024	0.828012	0.636909
**50%**	0.962920	0.907741	0.916154	0.905451	0.766414
**75%**	0.987306	0.954890	0.949695	0.943813	0.878401
**max**	1.000000	1.000000	0.976927	0.987676	0.913900

Table S9: Statistical summary of Precision, Recall, Accuracy, F-score, and MCC on 20 comparative genomics test data for TreasureIsland.

	Precision	Recall	Accuracy	F-Score	MCC
**mean**	0.998099	0.66912	0.816549	0.791256	0.683736
**std**	0.002964	0.165295	0.078247	0.118488	0.123808
**min**	0.993758	0.460823	0.745544	0.630908	0.567502
**25%**	0.996128	0.560187	0.755955	0.716978	0.588628
**50%**	1	0.650521	0.787893	0.787236	0.641204
**75%**	1	0.792481	0.876516	0.881574	0.776972
**max**	1	0.882978	0.926595	0.935576	0.859175

Table S10: Statistical summary of Precision, Recall, Accuracy, F-score, and MCC on 6 literature test data for IslandViewer.

	Precision	Recall	Accuracy	F-Score	MCC
**mean**	0.997641	0.478883	0.699898	0.636124	0.527399
**std**	0.003675	0.146042	0.10161	0.131124	0.117001
**min**	0.992308	0.311434	0.528242	0.474952	0.353072
**25%**	0.995155	0.366937	0.660759	0.535819	0.466205
**50%**	1	0.482958	0.730326	0.648781	0.549349
**75%**	1	0.543012	0.748064	0.703352	0.575676
**max**	1	0.704652	0.818949	0.824524	0.689684

Table S11: Statistical summary of Precision, Recall, Accuracy, F-score, and MCC on 6 literature test data for IslandPath Dimob.

	Precision	Recall	Accuracy	F-Score	MCC
**mean**	1	0.204859	0.553996	0.313323	0.271646
**std**	0	0.169161	0.055707	0.230444	0.165379
**min**	1	0	0.489312	0	0
**25%**	1	0.09267	0.515446	0.165735	0.200265
**50%**	1	0.205909	0.549358	0.340854	0.316717
**75%**	1	0.264649	0.579329	0.418123	0.350516
**max**	1	0.478183	0.642486	0.646987	0.473209

Table S12: Statistical summary of Precision, Recall, Accuracy, F-score, and MCC on 6 literature test data for SIGI HMM.

	Precision	Recall	Accuracy	F-Score	MCC
**mean**	1	0.226429	0.560047	0.353588	0.320506
**std**	0	0.135527	0.080844	0.171111	0.108267
**min**	1	0.066325	0.430562	0.1244	0.194896
**25%**	1	0.164463	0.538586	0.282462	0.255233
**50%**	1	0.198437	0.564586	0.330143	0.314516
**75%**	1	0.257023	0.58602	0.408652	0.346323
**max**	1	0.465685	0.677465	0.635451	0.506708

Table S13: Statistical summary of Precision, Recall, Accuracy, F-score, and MCC on 6 literature test data for Islander.

	Precision	Recall	Accuracy	F-Score	MCC
**mean**	0.75306	0.570324	0.704716	0.642011	0.39793
**std**	0.316267	0.271651	0.113373	0.298699	0.309797
**min**	0.135759	0.022974	0.501071	0.039298	-0.176955
**25%**	0.744039	0.619367	0.681418	0.699537	0.353387
**50%**	0.880677	0.67629	0.721528	0.758201	0.462629
**75%**	0.945899	0.706675	0.779522	0.796902	0.612083
**max**	0.955114	0.729194	0.817269	0.806581	0.660402

Table S14: Statistical summary of Precision, Recall, Accuracy, F-score, and MCC on 6 literature test data for Alien Hunter.

	Precision	Recall	Accuracy	F-Score	MCC
**mean**	0.963450	0.912426	0.926159	0.934079	0.859017
**std**	0.044112	0.091632	0.060427	0.049593	0.107435
**min**	0.876183	0.736344	0.807043	0.839462	0.648035
**25%**	0.970843	0.911428	0.934477	0.932169	0.873838
**50%**	0.974378	0.933870	0.939368	0.944674	0.879963
**75%**	0.983166	0.966175	0.961006	0.965095	0.921717
**max**	1.000000	0.989579	0.970108	0.975764	0.938708

Table S15: Statistical summary of Precision, Recall, Accuracy, F-score, and MCC on 6 literature test data for TreasureIsland.

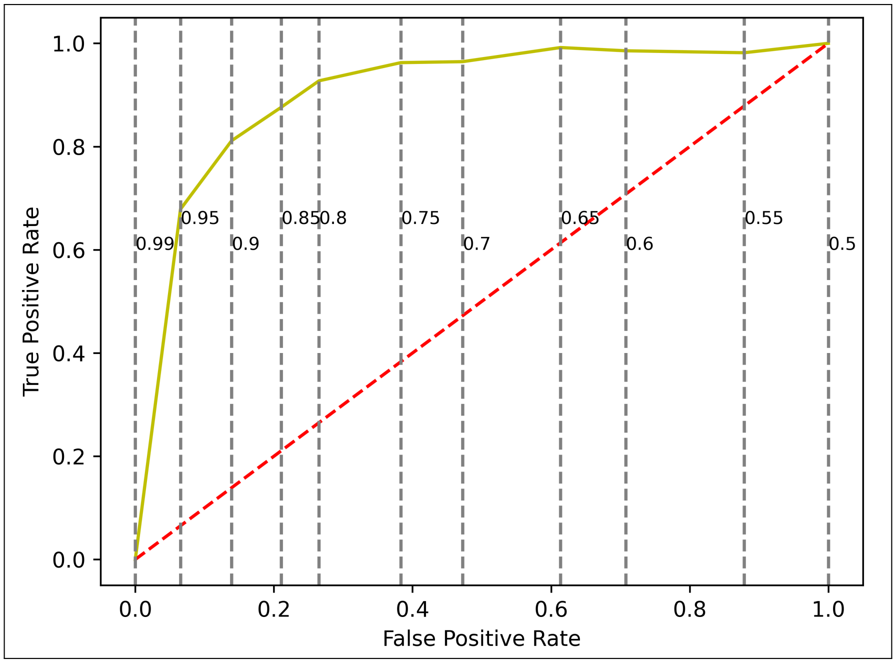

Figure S2: Receiver Operating Characteristic (ROC) curve showing the performance of TreasureIsland on changing upper threshold values on 70 training genomes. Vertical dashed lines: upper threshold; green line: AUC=0.89, Red line AUC=0.5

S5 Performance of TreasureIsland

To process one input sequence, TreasureIsland uses a linear computational time of $\mathcal{O}(N)$, and space complexity of $\mathcal{O}(N)$, where N is the length of the input DNA sequence in basepairs.

**Models**	**Input file**	**Average clock time taken to complete on 20 test genomes (in mins)**
IslandViewer4	Annotated files(eg. gbk)	49.75
IslandPath DIMOB	Annotated files(eg. gbk)	(Does not support Mac M1)
Sigi-HMM	Annotated files(eg. gbk)	**0.095**
Islander	Annotated files(eg. gbk)	(Software not avail)
Sigi-CRF	Uannotated files(eg. fasta)	9.05
AlienHunter	Uannotated files(eg. fasta)	6.2
Treasureisland	Uannotated files(eg. fasta)	**2**

Table S16: Average wall-clock time taken by IslandViewer4, IslandPath DIMOB, Sigi-HMM, Islander, Sigi-CRF, AlienHunter and Treasureisland to complete 20 test genomes. Test was run on Macbook M1, 16GB computer.

S6 Embedding model T-SNE (T-distributed stochastic neighbor embedding) visualization results

We used t-SNE as our dimensionality reduction technique to visually understand the effectiveness of our trained vectors. We have constrained our t-SNE results to the DBOW model as the overall classification results of the DBOW model is observed to be the most promising. Figures S3, S4, S5 showcases the t-SNE dimensionality reduction results of the vectors trained by the DBOW model. Figure 3 shows a much smaller set of training data comprising only Firmicutes or Bacillota phylum, which was used to train a DBOW model. This was trained for experimental purposes to understand the effectiveness of dataset size and variation on the DBOW model. Figure 4 shows the original training data vectors used to train the DBOW model. This also highlights the sequences which are GEI vs non-GEI to visually understand the separation in the vector space. Figure 5 shows the DNA context ID vectors trained in parallel to the DNA sequence IDs to visually understand the separation in the DNA context IDs.

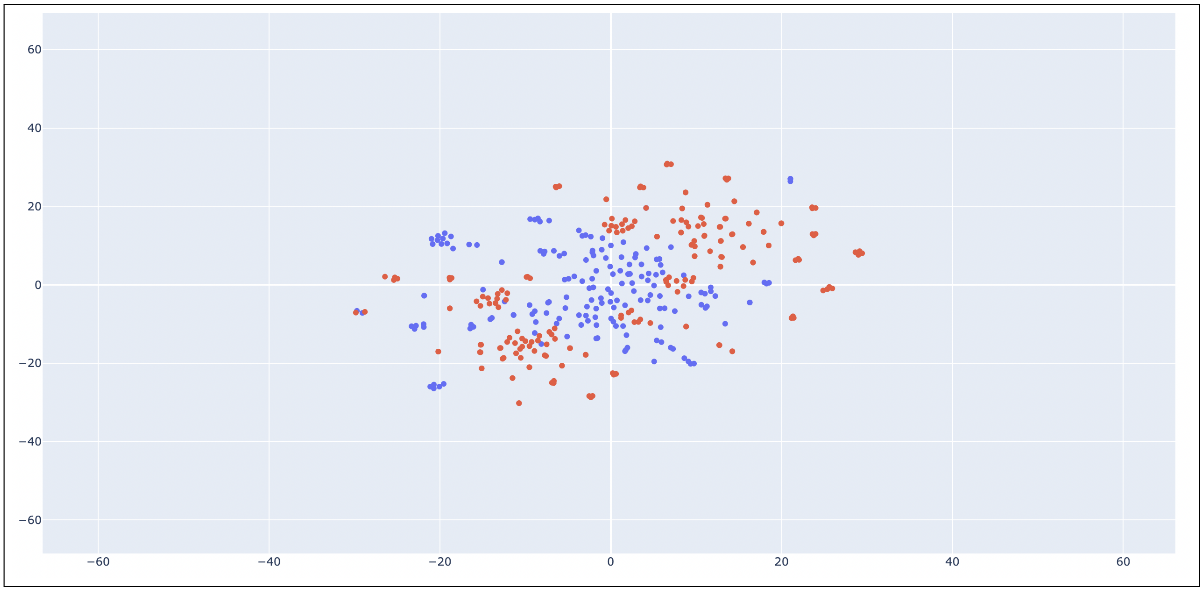

Figure S3: t-SNE (t-distributed Stochastic Neighborhood Embedding) visualization of the vectors from the DBOW model trained on 377 GEIs and non-GEIs from phylum Bacillota. ■ non-GEIs in Bacillota; ■ GEIs in Bacillota

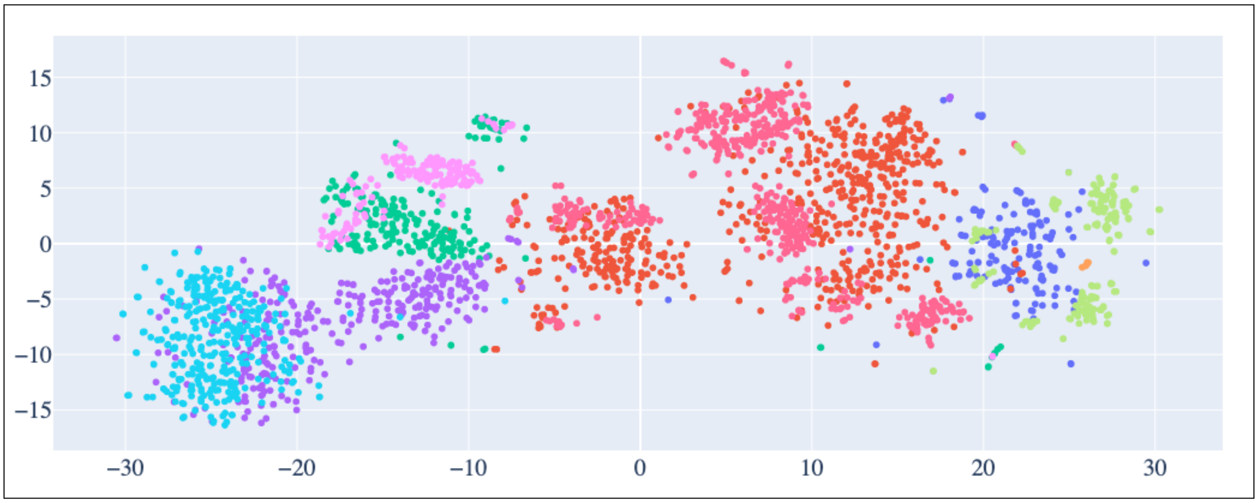

Figure S4: T-SNE (T-distributed Stochastic Neighborhood Embedding) visualization of the vectors from the DBOW model trained on 2569 GEIs and non-GEIs from phylum Pseudomonadota and Bacillota. ■ non-GEI in bacilli; ■ GEIs in bacilli; ■ non-GEIs in gammaproteobacteria; ■ GEIs in gammaproteobacteria; ■ non-GEIs in alphaproteobacteria; ■ GEIs in alphaproteobacteria; ■ non-GEIs in betaproteobacteria; ■ GEIs in betaproteobacteria; ■ GEIs in clostridia

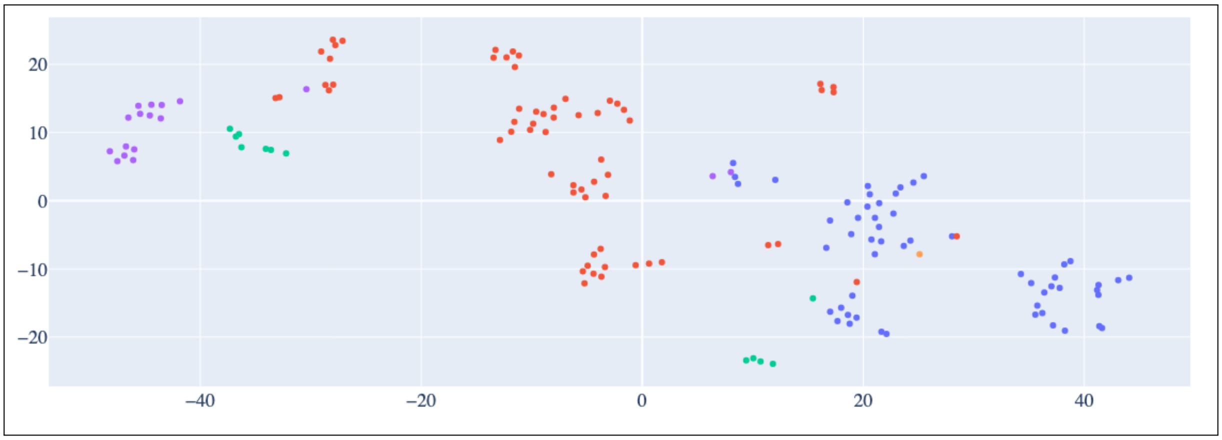

Figure S5: T-SNE (T-distributed Stochastic Neighborhood Embedding) visualization of the trained DNA context ID vectors from the DBOW model. ■ bacilli; ■ gammaproteobacteria; ■ alphaproteobacteria; ■ betaproteobacteria; ■ clostridia

S7 Benbow Dataset Taxonomy

**Phylum**	**Class**	**Order**	**Family**	**Species**	**Species (continued)**
Pseudomonadota	Alphaproteobacteria	Pasteurellales	Pseudomonadaceae	*Streptococcus equi*	*Pasteurella multocida*
Bacillota	Betaproteobacteria	Lactobacillales	Yersiniaceae	*Vibrio parahaemolyticus*	*Cronobacter sakazakii*
	Gammaproteobacteria	Bacillales	Neisseriaceae	*Vibrio campbellii*	*Rhizobium etli*
	Bacilli	Rickettsiales	Streptococcaceae	*Rickettsia conorii*	*Bradyrhizobium sp.*
	Clostridia	Pseudomonadales	Nitrobacteraceae	*Shigella sonnei*	*Yersinia enterocolitica*
		Vibrionales	Morganellaceae	*Streptococcus gallolyticus*	*Rickettsia massiliae*
		Hyphomicrobiales	Brucellaceae	*Vibrio vulnificus*	*Bacillus thuringiensis*
		Burkholderiales	Enterobacteriaceae	*Shigella dysenteriae*	*Streptococcus pneumoniae*
		Rhizobiales	Burkholderiaceae	*Yersinia pseudotuberculosis*	*Neisseria meningitidis*
		Enterobacterales	Phyllobacteriaceae	*Enterococcus faecium*	*Burkholderia cenocepacia*
		Eubacteriales	Shewanellaceae	*Rhodopseudomonas palustris*	*Staphylococcus haemolyticus*
		Alteromonadales	Moraxellaceae	*Burkholderia ambifaria*	*Proteus mirabilis*
		Moraxellales	Listeriaceae	*Clostridium perfringens*	*Bacillus cereus*
		Xanthomonadales	Xanthomonadaceae	*Yersinia pestis*	*Listeria innocua*
		Neisseriales	Rhizobiaceae	*Burkholderia lata*	*Bacillus subtilis*
		Thiotrichales	Pasteurellaceae	*Mesorhizobium loti*	*Rickettsia canadensis*
			Bacillaceae	*Staphylococcus saprophyticus*	*Burkholderia mallei*
			Clostridiaceae	*Streptococcus suis*	*Xanthomonas euvesicatoria*
			Staphylococcaceae	*Staphylococcus aureus*	*Shewanella pealeana*
			Bartonellaceae	*Pseudomonas aeruginosa*	*Staphylococcus argenteus*
			Vibrionaceae	*Shewanella halifaxensis*	*Escherichia coli*
			Rickettsiaceae	*Xanthomonas citri*	*Enterococcus faecalis*
			Francisellaceae	*Pseudomonas savastanoi*	*Burkholderia thailandensis*
			Enterococcaceae	*Salmonella enterica*	*Pseudomonas putida*
					Francisella tularensis
					Burkholderia orbicola
					Xanthomonas oryzae
					Brucella ovis
					Shewanella putrefaciens
					Xanthomonas campestris
					Burkholderia multivorans
					Pseudomonas syringae
					Brucella suis
					Shewanella baltica
					Streptococcus agalactiae
					Bartonella tribocorum
					Acinetobacter baumannii
					Shewanella sp.
					Staphylococcus epidermidis
					Listeria monocytogenes
					Haemophilus influenzae
					Vibrio cholerae
					Bacillus mycoides
					Ralstonia pseudosolanacearum
					Bacillus anthracis

Table S17: Taxonomic range covered by Benbow dataset.

S8 Prechecking prediction reliability

TreasureIsland initially checks for prediction reliability and will not take genomes too far removed from the taxa in Benbow. As mentioned in section *Phase 1: Model Construction under Computational Framework*) the input DNA sequence D is pre-processed initially by finding n non-overlapping segments d_i of DNA, $D = [d_1, d_2, d_3, ..., d_n]$ with probabilities $[p_1, p_2, ..., p_n]$ for any given segment d_i being a GEI is calculated. We then calculate the ratio of the number of segments R_{seg} with probabilities above the T_l (lower threshold, mentioned in Table 2) to the number of all segments.

$$
\begin{aligned}
&n_{pos} = \{d : d > T_l\} \\
&n = |D| \\
&R_{seg} = \frac{n_{pos}}{n}
\end{aligned}
$$

where n is the total number of segments in D, and n_{pos} is the number of segments with probabilities above the T_l lower threshold (mentioned in Table 2).

Segments with probabilities above the lower threshold would indicate segments belonging to the positive GEI category (class 1) and unclassified category (see Figure 3). If $R_{seg} > 0.7$, we consider it out of distribution and TreasureIsland outputs "cannot confidently predict genomic islands for this sequence". A high ratio indicates the model cannot identify negative regions in the input. This implies that the model fails to find the core genome in the input, and the input is, therefore, likely from an taxon that is outside the scope of the training set. The threshold of 0.7 was determined by calculating the average ratio across more than 50 genomes outside the taxonomic phylum covered in the Benbow dataset.

References

[1] Jörg Hacker and James B Kaper. "Pathogenicity islands and the evolution of microbes". In: *Annual Reviews in Microbiology* 54.1 (2000), pp. 641–679.

[2] Tomas Mikolov et al. "Efficient estimation of word representations in vector space". In: *arXiv preprint arXiv:1301.3781* (2013).

[3] Patrick Ng. "dna2vec: Consistent vector representations of variable-length k-mers". In: *arXiv preprint arXiv:1701.06279* (2017).

[4] Juan Ramos et al. "Using tf-idf to determine word relevance in document queries". In: *Proceedings of the first instructional conference on machine learning*. Vol. 242. 1. Citeseer. 2003, pp. 29–48.
